# The Effect of the Congruence between Job Characteristics and Personality on Job Crafting

**DOI:** 10.3390/ijerph17010052

**Published:** 2019-12-19

**Authors:** Mihee Kim, Seung Ik Baek, Yuhyung Shin

**Affiliations:** 1 Korea Institute for Defense Analyses, Seoul 02455, Korea; mihee@kida.re.kr; 2 School of Business, Hanyang University, Seoul 04763, Korea; sbaek@hanyang.ac.kr

**Keywords:** job characteristics, job crafting, personality

## Abstract

This study examined the effect of the fit between personality (i.e., openness to experience) and core job characteristics (i.e., skill variety, task significance, and task identity) on job crafting. We collected survey data from 200 college students who were assigned a team project during the semester. Using polynomial regression analysis, we tested the effects of the fit between personality and job characteristics on job crafting. The results revealed that a high level of openness to experience was significantly associated with a high level of job crafting (i.e., task, relational, and cognitive crafting). Furthermore, when both openness to experience and job characteristics were congruent at a high level, the tendency to proactively perform one’s tasks was also high. These findings enhance our understanding of the effect of the fit between openness to experience and three core job characteristics on job crafting.

## 1. Introduction

Fast-changing business environments and recent advancements in information technology have resulted in a gradual rallying of organizational members’ proactive attitudes and behaviors. To achieve organizational success, it is necessary for members to demonstrate proactive attitudes and behaviors when they perform their tasks. Therefore, organizations should dispense with conventional approaches to job design and promote job crafting, which refers to a member-oriented job redesign process. Job crafting refers to the process by which members’ proactive behaviors transform the characteristics and limitations of their jobs [1,2]. In other words, instead of performing a given task in a passive manner, job crafting allows members to actively create and facilitate changes when they perform their tasks. Changes in contemporary working environments (e.g., the provision of resources that are voluntarily utilized by organizational members and the increasing importance of job-oriented human resource practices) underscore the importance of researching job crafting. Many research studies have found that job crafting reduces job burnout [3] and has a positive effect on job satisfaction [4] and performance [5,6] and innovative behaviors [7]. However, existing literature on the factors that facilitate job crafting is limited. While researchers have identified individual characteristics (e.g., a proactive personality, narcissism, Machiavellianism, and boundaryless career orientation [8,9,10]) as the antecedents of job crafting, few studies have assessed the effect of the interaction or fit between individual and job characteristics on job crafting. To address these gaps in the existing literature, the present study examined the effect of the fit between individual and job characteristics on job crafting within the framework of person-job (P-J) fit theory.

Person-environment fit theory [11,12] maintains that a good fit between individual and environmental characteristics contributes to positive attitudes and better performances. Of the different types of person-environment fit, P-J fit pertains to the interaction between a person and his or her job; specifically, it refers to the extent to which there is a fit between an individual’s abilities and the characteristics that are required by a job (i.e., demands-abilities fit) or a fit between an individual’s needs and the resources that a job offers (i.e., needs-supplies fit) [13]. In this study, we propose needs-supplies fit as a precondition of job crafting. Using P-J fit theory as a theoretical framework, we attend to one individual characteristic, namely, openness to experience, and three job characteristics, namely, skill variety, task significance, and task identity. Of the Big Five personality traits (i.e., conscientiousness, agreeableness, neuroticism, openness to experience, and extraversion), openness to experience is known as the strongest predictor of creativity in numerous studies [14,15,16,17,18,19,20]. Individuals who are open to experience actively seek out new possibilities and pursue challenges that transform their work experience [21], which is a critical aspect of job crafting [6]. Therefore, job crafting requires a higher degree of openness to experience than other personality traits. For this reason, we focus on openness to experience as a key personality trait that predicts job crafting. Adopting the needs-supplies perspective of P-J fit, we further argue that when individuals have needs for openness to experience, they seek job characteristics that can fulfil those needs. Skill variety, task significance, and task identity are job characteristics that can satisfy individuals’ needs for openness to experience. As individuals who are open to experience seek diverse opportunities and experiences, they prefer tasks that allow them to use of various skills (i.e., high skill variety), have an impact on other organizational members (i.e., high task significance), and experience the entire process of a project (i.e., high task identity). Therefore, skill variety, task significance, and task identity are job characteristics that are relevant to openness to experience in assessing needs-supplies fit. Furthermore, job characteristics theory [22] proposes that these three job characteristics are related to feelings of meaningfulness that members experience when performing a task. Because feelings of meaningfulness are closely related to job crafting [23,24], skill variety, task significance, and task identity are crucial job aspects that are associated with job crafting. Furthermore, this study was conducted within the context of a team project, which required members to perform a common task in accordance with stipulated procedures and within a certain deadline. Therefore, since team projects offer limited opportunities to be autonomous and receive feedback, we believed that the three aforementioned job characteristics may be more readily observable in such a context. Accordingly, we focused on these three core job characteristics. To examine the effect of the fit between individual (i.e., openness to experience) and job characteristics (i.e., skill variety, task significance, and task identity) on job crafting, the present study examined the independent effects of the two factors on job crafting and used polynomial regression analysis and a response surface test to investigate the fit.

### 1.1. Theoretical Background and Hypothesis Development

#### 1.1.1. Job Crafting

Wrzesniewski and Dutton [25] defined job crafting as the process by which organizational members make their work more meaningful by transforming tasks in an autonomous and proactive manner. In contradistinction to the traditional focus of business administration (i.e., a top-down job design), job crafting adopts a bottom-up approach to individual job redesign. This permits members to not only redefine and reorganize, but also voluntarily lead and proactively perform their jobs [6,26]. Despite being a form of proactive behavior, job crafting differs from proactive behavior in that it involves coping strategies to tackle the changing demands of the work context [6].

Using the criteria that Wrzesniewski and Dutton [25] have proposed, job crafting can be categorized into three primary types based on the aspects of a job that have been changed [27]. First, task crafting is an attempt to change the physical aspects of tasks (e.g., changing a preexisting method of performing a task and expanding or diminishing the scope of a task) in accordance with one’s own needs. For example, utilizing the various features of social media services to promote more effective communication in the workplace can enhance an organizational member’s familiarity with and interest in social media services. Subsequently, he or she may accept the task of utilizing and managing these services for the company [28]. Second, relational crafting aims to improve and transform business relationships by changing the frequency or quality of interactions among organizational members in the workplace. For example, marketing managers often share closer relationships with salespersons than with others because the strong interrelatedness of their work enhances their mutual understanding [29]. Third, cognitive crafting refers to the process of transforming the boundaries of one’s perceptions (e.g., reinterpreting and appreciating the objective or meaning of one’s job). For instance, a janitorial staff member who works in a hospital may realize that his or her job is not to merely provide cleaning services. Instead, he or she may realize that, by maintaining the cleanliness of the hospital, he or she is contributing toward patient recovery [25].

#### 1.1.2. The Main Effects of Openness to Experience and Job Characteristics on Job Crafting

Innovative and creative thinking has become increasingly important to organizations because these characteristics help members cope with the rapidly changing demands of contemporary work environments. In this regard, many researchers have examined many related personality traits, especially openness to experience, which is characterized by imaginative, original, and independent thinking; curiosity; adventure-seeking tendencies; and a preference for novel stimuli [30,31]. Hildenbrand and colleagues [32] contend that openness to experience is a key resource that enables individuals to adopt efficient coping strategies through active selection and implementation. Furthermore, since there are individual differences in how resources are evaluated, the evaluation process is contingent on the fit between the individual and his or her resources [33,34]. Therefore, we predicted that openness to experience would be more strongly related to job crafting when compared to the other traits that are subsumed by the Big Five model of personality traits.

Individuals who are highly open to experience easily accept new perspectives. Moreover, their preference for new experiences allows them to be innovative, creative, and tolerant of changes, rather than passively adapt to their jobs [35]. In addition, across a wide range of contexts, members who are highly open to experience pay attention to others and have extremely high levels of cognitive and affective empathy [36]. Therefore, when members with high levels of openness to experience perform a job, they readjust their workloads and the scope of their tasks based on their own criteria because they tend to be independent thinkers and proactive workers. In addition, such members share favorable relationships with others by engaging in forthright interactions [37]. They are also more likely to easily acquire the cognitive and psychological resources that are needed to perform a job. Moreover, given that an attitude of acceptance toward new perspectives requires openness to experience, this characteristic may encourage workers to view issues from different angles, thereby providing them with an opportunity to reflect upon the meaningfulness or essence of their work. Accordingly, the following hypothesis was formulated:

**Hypothesis** **1.** *Openness to experience will be positively associated with job crafting*.

Hackman and Oldham’s [22] job characteristics theory proposes that there are five core job characteristics. First, skill variety refers to the extent to which complex and diverse skills or talents are used to perform a job. Second, task significance refers to the extent to which a job has a substantial impact on other organizational members’ work, as well as the work and life of related individuals who do not belong to the organization. For example, an individual’s realization that his or her job has a major impact on others’ happiness may reinforce the perceived meaningfulness of his or her job. Third, task identity refers to the extent to which an individual member is involved in the entire process of a task or project. Fourth, autonomy refers to the level of freedom, independence, and leeway that is afforded to individuals, when they have to make decisions about work procedures or plans. Fifth, feedback refers to the amount of direct and clear information that members receive about the outcomes of their job performance; feedback facilitates the acquisition and development of knowledge about job outcomes.

Job characteristics theory further asserts that core job characteristics help organizational members experience adaptive psychological states (e.g., meaningfulness, and responsibility toward and awareness about job outcomes), which in turn enhance work motivation and promote positive attitudes and behaviors [38]. In particular, each core job dimension has a differential, albeit direct, influence on critical psychological states. A person whose job entails a high level of skill variety, task significance, and task identity will experience meaningfulness [38]. High levels of autonomy cause organizational members to believe that they are responsible for job outcomes, and high levels of feedback-seeking tendencies help organizational members acquire information about their performance. Because job crafting involves attempts to improve or transform one’s jobs in such a manner that it fosters greater meaningfulness [25], we posited that the three aforementioned characteristics may be more relevant to job crafting than the other two characteristics. Prior findings have demonstrated that job crafting not only leads to work meaningfulness, but is also influenced by work meaningfulness [23,24], which suggests that job characteristics that can inculcate meaningfulness can also promote job crafting. Accordingly, the three job characteristics (i.e., skill variety, task significance, and task identity) were expected to be more directly associated with job crafting than other two characteristics. Specifically, high levels of skill variety broaden the scope of job performance, reduce the monotony of a job, and enable workers to demonstrate a better job performance by acquiring the requisite skills [39]. Therefore, such workers may create new methods of performing their jobs or independently change the scope of their jobs. In addition, greater opportunities to acquire skills and cognitive expansion are likely to help workers contemplate the meaning of their work and the purpose of the organization. Jobs that foster a high level of task significance have an impact on not only the organization, but also external stakeholders. Therefore, we predicted that those who perform such jobs will experience greater meaningfulness as a result of their enhanced faith in their capabilities. Additionally, since their altruistic motives are reinforced by their willingness to take responsibility for significant tasks, they may be more proactive in performing their tasks. Those who perform tasks that entail a high level of task identity are likely to realize that their jobs are resonant with the objectives of the organization as a whole, and this may increase their sense of ownership over job outcomes [39]. In addition, as they become more confident about their influence and job skills [40], they are likely to use unique methods and procedures of performing their jobs. Performing a job that entails a high level of task identity will increase interactions with superiors, advisors, and coworkers because these exchanges are essential to successful task completion. Performing a job that entails a high level of task identity is also likely to help workers experience a sense of meaningfulness, and this in turn may offer them the opportunity to reflect upon and reinterpret the value of their work. Therefore, given these considerations, the following hypotheses were proposed:

**Hypothesis** **2.** *Skill variety will be positively associated with job crafting*;

**Hypothesis** **3.** *Task significance will be positively associated with job crafting*;

**Hypothesis** **4.** *Task identity will be positively associated with job crafting*.

#### 1.1.3. The Effect of the Congruence between Openness to Experience and Job Characteristics on Job Crafting

This study examined the interactions between personality traits and job characteristics from the perspective of P-J fit theory. In the present study, we conceptualize P-J fit as the fit between a person’s demands and job characteristics (i.e., needs-supplies fit) [11,13,41]. P-J fit is significantly related to an individual’s job situation or attitudes (e.g., job satisfaction, stress, and motivation) [13,42]. In addition, P-J fit has been found to directly improve job performance and organizational citizenship behaviors, and it is significantly associated with proactive behaviors [43,44,45]. According to the six areas of the worklife model [45], workload, control, rewards, community, fairness, and value congruence are six critical conditions of employee engagement at work. From this perspective, the lack of fit between a person and his/her job is a main cause of job stress and burnout [44]. Moreover, this model theorizes that workplace congruence (i.e., P-J fit) serves to expand psychological resources that enable employees to cope with job demands [45]. Grounded in this theorizing, we assessed the fit between individuals’ personality traits and job characteristics as a precondition of job crafting. In sum, it was expected that the congruence between demand (i.e., the task performer’s openness to experience) and supply (i.e., job characteristics) will promote job crafting.

Members with a high level of openness to experience think proactively, are open to changes and experiences, and pursue learning opportunities, even under difficult and complex circumstances [35]. Furthermore, they care about others (including their superiors and coworkers) and possess excellent social skills (i.e., approaching and interacting with others, and exhibiting cognitive and affective empathy). Consequently, they engage in creative and original thinking to solve interpersonal and cognitive problems [36]. Skill variety offers such individuals the opportunity to learn various skills while they perform their tasks, and this in turn offers them new experiences and stimulation. In sum, when the demand for openness to experience and supply of skill variety are both high, workers are likely to perform their tasks proactively. In addition, task significance satisfies the need for cognitive challenges among members with high levels of openness to experience; therefore, workers are expected to engage in high levels of job crafting when levels of openness to experience and task significance are both high. Finally, task identity allows members with high levels of openness to better understand the overall mission and vision of the organization and provides them with an opportunity to interact with superiors and coworkers and experience a sense of meaningfulness. Therefore, high levels of openness to experience and task identity were expected to promote proactive job crafting. Based on the preceding discussion, the following hypotheses, which pertain to the interaction between openness to experience and job characteristics, were proposed:

**Hypothesis** **5.** *Individuals will engage in higher levels of job crafting when there is congruence between their levels of openness to experience and skill variety than when there is incongruence between these two characteristics*;

**Hypothesis** **6.** *Individuals will engage in higher levels of job crafting when there is congruence between their levels of openness to experience and task significance than when there is incongruence between these two characteristics*;

**Hypothesis** **7.** *Individuals will engage in higher levels of job crafting when there is congruence between their levels of openness to experience and task identity than when there is incongruence between these two characteristics*.

## 2. Method

### 2.1. Sample

To test the study hypotheses, a survey was conducted among 200 students who were enrolled in courses on business administration at a university in Seoul. Among the courses that were offered during the fall semester of 2016, the three courses that required students to actively participate in a team project were selected. The team project was designed to analyze a business case whose purpose was to provide an opportunity for students to improve their business insight. At the beginning of the semester, students were randomly assigned to autonomous teams in which they voluntarily divided their roles and responsibilities. As a part of each course, the students were required to prepare for the team project for approximately 30 min after class hours. Students spent this time discussing how they should prepare for the assigned project by delegating tasks, coordinating schedules, and engaging in lively interactions. In order to undertake this project, students were required to possess (a) the ability to conduct a literature review, collect and analyze various types of data, and prepare a proposal, as well as (b) writing and presenting skills. Two hundred questionnaires were distributed, but only 177 returned questionnaires contained usable data (a response rate of 88.5 %). Specifically, 23 questionnaires contained missing responses. On average, there were 5.2 members in each team. The sample consisted of 75 female (51%) and 72 male (49%) students. Sixty-six (37%), 53 (29%), and 43 (24%) students were sophomores, juniors, and seniors, respectively. Their majors varied: there were 146 (82%) students in business administration/finance, 15 (8%) in social sciences, 10 (6%) in science and engineering, and 6 (3%) in liberal arts.

### 2.2. Measures

In this study, survey items were constructed using Brislin’s [46] back-translation procedure, and all variables were assessed with multiple items on a five-point Likert type scale (1 = *strongly disagree*, 5 = *strongly agree*). To ensure the reliability and validity of our measures, following Costello and Osborne’s [47] recommendation, we eliminated cross-loading items to improve model fit. More precisely, items with a factor loading lower than 0.40 were eliminated from our measurement scales [48]. This practice has been commonly used in prior psychology research [49,50]. The measurement items are presented in Table 1.

#### 2.2.1. Openness to Experience

To measure openness to experience, we used MINI-IPIP scales [51] which consisted of 20 items measuring big-five personalities. This abbreviated scale of big-five personalities has been widely used in prior research [52,53]. In line with previous research that used a shortened scale of openness to experience [53,54,55], of the four items of openness to experience, an item with a poor factor loading was eliminated. Examples of the retained items are “I have a vivid imagination” and “I am interested in abstract ideas.”

#### 2.2.2. Job Characteristics

The job characteristics that were assessed in this study, namely, skill variety, task significance, and task identity, were assessed using the Work Design Questionnaire, which was developed by Morgeson and Humphrey [56]. The items of this assessment were modified in accordance with the project that had been assigned to the students of each course. Specifically, four, three, and three items measured skill variety (α = 0.81), task significance (α = 0.86), and task identity (α = 0.78), respectively. Examples of items that were posed to each group are as follows: “My role requires a diverse set of complex skills that are necessary to complete the team assignment” (skill variety); “My role bears significance to the successful completion of the team assignment” (task significance); and “The objectives and outcomes of my role are clearly delineated when we work on the team assignment” (task identity).

#### 2.2.3. Job Crafting

In this study, the three dimensions of job crafting, namely, task, relational, and cognitive crafting, were measured, and the proposed hypotheses were tested in relation to each dimension. Specifically, the fifteen-item Job Crafting Questionnaire, which was developed by Slemp and Vella-Brodrick [28], was modified in accordance with the context within which job crafting was assessed (i.e., team assignments). Consistent with prior research that relied on the abbreviated scales of job crafting [4,57], items with low factor loadings were excluded; thus, participant responses to 12 items were analyzed. Four, five, and three items assessed task (α = 0.76), relational (α = 0.82), and cognitive crafting (α = 0.76), respectively. Examples of the items that were used to assess these dimensions are as follows: “I have tried a new approach to get the assignment done well”; “I have tried to organize or actively participate in social activities and meetings that are related to the team assignment”; and “I have thought about the kind of positive impact that my role in the group assignment will have on my college life.”

### 2.3. Analytical Strategy

In accordance with Edwards and Parry’s [58] recommendations, the study hypotheses were tested using polynomial regression analysis and the three-dimensional surface test, which have been widely used in research on person-environment fit. This method allows researchers to precisely examine the interactive relationship or effect of the congruence between two variables, namely, the person and environment, on the outcome variable [58]. Polynomial regression equations contain high-order terms for person and environment components (e.g., squares terms for each component and its product) [13]. Accordingly, our polynomial regression equation consisted of control variables (i.e., two dummies for class and four dummies for grades), a predictor (i.e., the personality trait of openness to experience), and the dependent variable (i.e., three job characteristics: skill variety, task significance, and task identity). To test the hypotheses, each type of job crafting was regressed on the control variables and five polynomial terms, which were as follows: personality and its square term; job characteristics and its square term; and the product term of personality and job characteristics. To reduce multicollinearity among variables, all variables’ scales were mean-centered prior to all analyses.

According to Edwards and Parry [58], if any high-order term (i.e., openness to experience^2^, openness to experience × job characteristics, and job characteristics^2^) is significant, the effect of the congruence between two person and environment components on the outcomes can be illustrated using a three-dimensional graph by conducting a response surface test using regression coefficients. In particular, we examined the curvature along the incongruence line (openness to experience [X] = job characteristics [Y]) on a three-dimensional graph. In order for the congruence effect to be observed, there should be a significant negative curvature along the incongruence line on a three-dimensional graph [59]. In other words, the level of job crafting should be higher when there is congruence between individual and job characteristics (openness to experience [X] = job characteristics [Y] line) than when there is incongruence.

## 3. Results

Using AMOS version 18.0, a confirmatory factor analysis (CFA) was conducted to confirm the factor structure of the measures used in the study and verify the independence of the variables that they assessed. The model fit of a seven- (i.e., personality trait: openness to experience; three job characteristics; and the three types of job crafting) and three-factor model was compared (i.e., three job characteristics and the three types of job crafting). The results suggested that the seven-factor model (χ² = 549.4, df = 254, CFI = 0.89, RMSEA = 0.06) was a better fit for the data than the three-factor model (χ² = 867.32, df = 272, CFI = 0.685, RMSEA = 0.12) (Δχ² = 318.02, Δdf = 18, *p* < 0.001).

Descriptive statistics and intercorrelations between the study variables are presented in Table 2. As predicted, there were significant correlations (r = 0.16–0.52) between openness to experience and each of the three job characteristics and types of job crafting.

To test the research hypotheses, polynomial regression analysis was conducted. First, the control variables (i.e., course and grade dummies) were entered in all the analyses in an identical manner. Next, the linear and high-order terms (i.e., personality^2^, personality × job characteristic, and job characteristic^2^) were entered into each analysis. We examined whether the model significantly predicted the dependent variable and whether any high-order term was significant. Based on the results, the effect of the congruence between personality and job characteristics on job crafting was investigated by conducting a response surface test.

### 3.1. Analytical Results for Task Crafting

The results of polynomial regression analysis, which was conducted to examine the effect of the fit between openness to experience and job characteristics on task crafting, are presented in Table 3. In Model 1, the control variables were entered. In Model 2, the main effects were examined by entering high-order terms for each job characteristic in a sequential order after entering openness to experience and the three job characteristics.

First, with regard to the main effects, openness to experience (β = 0.21, *p* < 0.01), skill variety (β = 0.35, *p* < 0.001), and task significance (β = 0.19, *p* < 0.01) predicted task crafting positively and significantly (see Model 2); task identity did not emerge as a significant predictor (β = 0.06, *p* = n.s.).

With regard to openness to experience and skill variety, the regression equation that included the polynomials significantly predicted task crafting (∆R^2^ = 0.33, *p* < 0.001), but none of the three polynomial terms (openness to experience = 0.02, p = n.s.; openness to experience × skill variety = −0.10, p = n.s.; skill variety^2^ = 0.07, *p* = n.s.) significantly predicted task crafting (see Model 3). The response surface test results revealed that the slope of Y = X was significant and that the curvature of Y = −X was not significant, which suggested that the congruence did not have a significant effect on task crafting.

**Figure 1:**
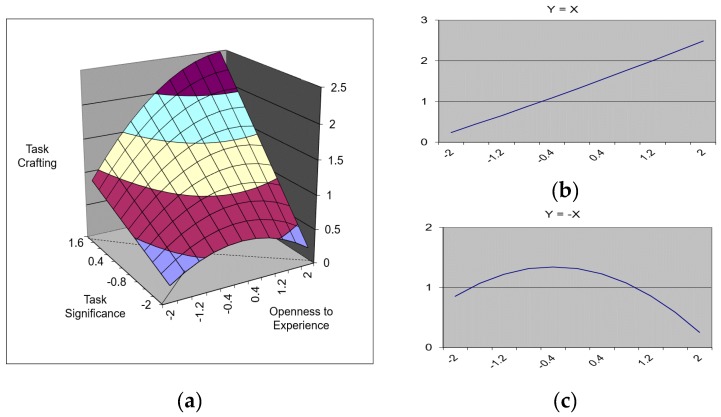
A surface graph depicting the effect of the fit between openness to experience and task significance on task crafting. (**a**) The three-dimensional relationship between openness to experience, task significance, and task crafting. (**b**) The slope of Y = X line. (**c**) The curvature of Y = −X line.

Model 4 examined the congruence between openness to experience and task significance. The results revealed that the regression equation which included polynomials significantly predicted task crafting (∆R^2^ = 0.27, *p* < 0.001); further, the squared term of openness to experience, which was one of the three polynomials, was also statistically significant (β = −0.12, *p* < 0.10). Based on these results, the response surface test was conducted, and the results showed that the slope of Y = X and curvature of Y = X were significant. The graph for the effect of the fit is shown in Figure 1. The three-dimensional graph (Figure 1a), which is presented on the left side of the figure, shows the effect of the fit between openness to experience and task identity. To examine the effect of the fit in greater detail, the three-dimensional graph was cut along the Y = X and Y = −X axis, as shown on the right side of the graph. The Y = X graph (Figure 1b) shows that the level of job crafting was highest when openness to experience and task significance were both high (i.e., [5,5] rather than [1,1]). With regard to the Y = X graph (Figure 1c), the curvature was negative, and the level of task crafting was highest when openness to experience and task significance were congruent (i.e., [3,3] rather than [1,5] or [1,5]). The trends that emerged for openness to experience and task identity were the same as those that emerged for skill variety. The regression equation that included the polynomial terms significantly predicted task crafting (∆R^2^ = 0.21, *p* < 0.001), but the three high-order terms (openness to experience^2^ = 0.06, *p* = n.s.; openness to experience × task identity = 0.02, *p* = n.s.; task identity^2^ = 0.00, *p* = n.s.) and response surface test results did not support the hypothesis that pertained to the effect of the fit between openness to experience and task identity (see Model 5).

### 3.2. Analytical Results for Relational Crafting

To examine the effect of the congruence between openness to experience and job characteristics on relational crafting, the same analyses (i.e., polynomial regression analysis and the response surface test) that have been described in the preceding section were conducted; the corresponding results are shown in Table 4.

First, with regard to the main effects (see Model 7), skill variety (β = 0.27, *p* < 0.01) predicted relational crafting significantly and positively, and the other variables failed to emerge as significant predictors (openness to experience: β = 0.06, *p* = n.s.; task significance: β = 0.11, *p* = n.s.; task identity: β = 0.08, *p* = n.s.).

**Figure 2:**
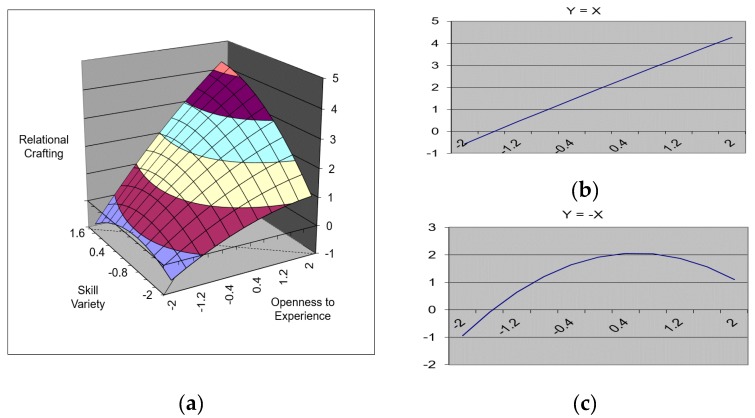
A surface graph depicting the effect of the fit between openness to experience and skill variety on relational crafting. (**a**) The three-dimensional relationship between openness to experience, skill variety, and relation crafting. (**b**) The slope of Y = X line. (**c**) The curvature of Y = −X line.

As can be inferred from Model 8, the linear and high-order terms significantly predicted relational crafting (∆R^2^ = 0.18, *p* < 0.001), and the interaction term between openness to experience and skill variety (β = 0.18, *p* < 0.05) and the squared term of skill variety (β = −0.15, *p* < 0.05) were statistically significant. The results of the response surface test revealed that the slope of Y = X (Figure 2b) was significant and that the curvature of Y = −X (Figure 2c) entailed a significant and negative value. The corresponding three-dimensional graph results are shown in Figure 2a. Specifically, when openness to experience and skill variety were congruent at a higher level, the level of relational crafting was also higher. 

**Figure 3:**
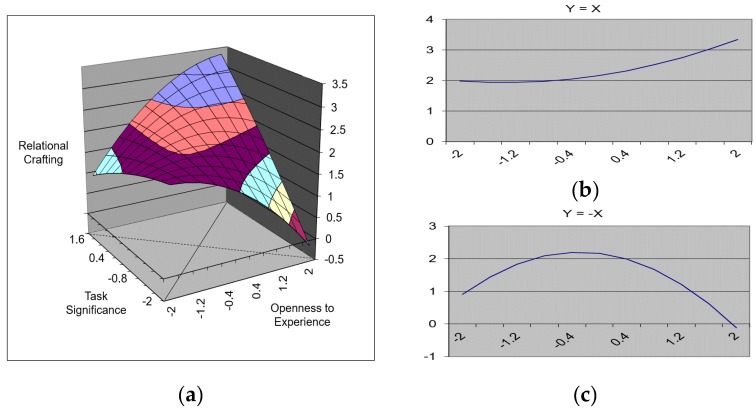
A surface graph depicting the effect of the fit between openness to experience and task significance on relational crafting. (**a**) The three-dimensional relationship between openness to experience, task significance, and relational crafting. (**b**) The slope of Y = X line. (**c**) The curvature of Y = −X line.

The results that emerged for Model 9 revealed that the fit between openness to experience and task significance significantly predicted relational crafting (∆R^2^ = 0.14, *p* < 0.001). Furthermore, among the polynomials, the squared term of openness to experience was also significant (β = −0.15, *p* < 0.10). The results of the corresponding response surface test revealed that the slope of Y = X (Figure 3b) entailed a significant and positive value and that the curvature of Y = −X (Figure 3c) entailed a significant and negative value. Consistent with our predictions, the results presented in Figure 3a indicate that, when openness to experience and task significance were congruent at a higher level, the level of relational crafting also increased.

However, with regard to openness to experience and task identity, only the linear term of task identity significantly predicted relational crafting, and none of the three polynomials was significant (openness to experience^2^ = −0.07, *p* = n.s.; openness to experience × task identity = 0.04, *p* = n.s.; task identity^2^ = −0.04, *p* = n.s.). Accordingly, the response surface test results revealed that the curvature of Y = −X was not statistically significant (see Model 10).

### 3.3. Analytical Results for Cognitive Crafting

The effect of the fit between openness to experience and job characteristics on cognitive crafting was examined using the same procedures that were used in the previously reported analyses. The results of the polynomial regression analysis are depicted in Table 5.With regard to the main effects, skill variety (β = 0.32, *p* < 0.001) and task identity (β = 0.17, *p* < 0.05) predicted cognitive crafting positively and significantly, but the other variables did not emerge as significant predictors (openness to experience: β = 0.07, *p* = n.s.; task significance: β = −0.05, *p* = n.s.) (see Model 12).After control variables were included, the polynomials were entered to examine the effect of the fit between openness to experience and skill variety (∆R^2^ = 0.19, *p* < 0.001). The results showed that the interaction (β = −0.11, *p* = n.s.) and squared terms (β = −0.13, *p* = n.s.; β = −0.07, *p* = n.s.) were not significant. Moreover, the response surface test results revealed that the curvature of Y = −X, which is a measure of the effect of the fit, was not significant (see Model 13 in Table 4). In contrast, with regard to openness to experience and task significance, Model 14 included the polynomials that significantly predicted cognitive crafting (∆R^2^ = 0.07, *p* < 0.05), but only the linear terms of openness to experience (β = 0.15, *p* < 0.10) and task significance (β = 0.14, *p* < 0.10) significantly predicted cognitive crafting. In other words, none of the polynomials that could have verified the effect of the fit were statistically significant (β = 0.01, *p* = n.s.; β = 0.08, *p* = n.s.; β = −0.00, *p* = n.s.). Accordingly, the response surface test results also showed that the curvature of Y = −X, which is a measure of the effect of the fit, was not statistically significant (a_1_ = 0.55, *p* < 0.001; a_4_ = 0.16, *p* = n.s).

**Figure 4:**
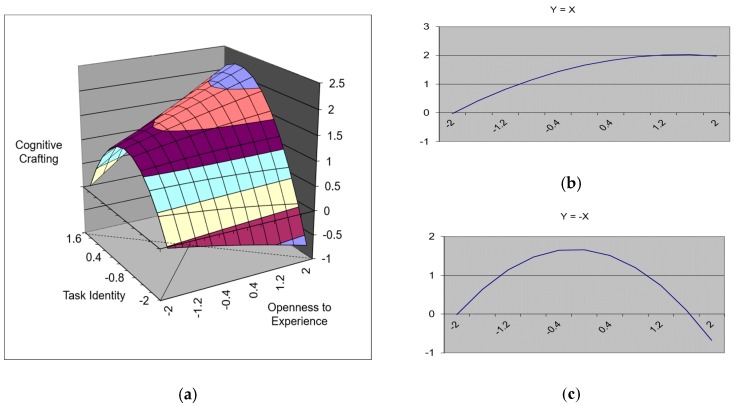
A surface graph of the effect of the fit between openness to experience and task identity on cognitive crafting. (**a**) The three-dimensional relationship between openness to experience, task identity, and cognitive crafting. (**b**) The slope of Y = X line. (**c**) The curvature of Y = −X line.

In contrast, with regard to openness to experience and task identity, the regression equation that included polynomials significantly predicted cognitive crafting (∆R^2^ = 0.16, *p* < 0.001). Among the polynomials, the squared term of task identity (β = −0.21, *p* < 0.01) also significantly predicted cognitive crafting (see Model 15 in Table 4). The response surface test results also showed that both the slope of Y = X (Figure 4b), which entailed a positive value (a_1_ = 0.50, *p* < 0.001), and the curvature of Y = −X (Figure 4c), which entailed a negative value (a_4_ = −0.50, *p* < 0.01), were significant. The effect of the fit between openness to experience and task significance can be examined in greater detail by inspecting the three-dimensional graph that is presented in Figure 4a. It shows that, when openness to experience and task identity were congruent at a higher level, the level of cognitive crafting was also higher.

Of the three types of job crafting that were examined, openness to experience only predicted task crafting, and its coefficient was positive (β = 0.21, *p* < 0.01); these results offer partial support to Hypothesis 1. Task significance, which is a job characteristic, also only had a statistically significant effect on task crafting (β = 0.19, *p* < 0.01); this result also offers partial support to Hypothesis 3. Task identity only predicted cognitive crafting (β = 0.17, *p* < 0.05), and this result partially supported Hypothesis 4. On the other hand, skill variety had a positive effect on all three types of job crafting (β = 0.35, *p* < 0.001; β = 0.27, *p* < 0.01; β = 0.32, *p* < 0.001); these results support Hypothesis 2. Additionally, the results of tests of hypotheses that pertained to the fit between openness to experience and each job characteristic were as follows: (a) the fit between openness to experience and skill variety only had a significant effect on relational crafting (i.e., partial support for Hypothesis 5); (b) the fit between openness to experience and task significance had a significant effect on both task and relational crafting (i.e., partial support for Hypothesis 6); and (c) the fit between openness to experience and task identity only had a significant effect on cognitive crafting (i.e., partial support for Hypothesis 7).

## 4. Discussion

### 4.1. Theoretical Implications

The present study investigated the effects of the fit between personality traits and job characteristics on job crafting. The results demonstrated the distinct effects of the three job characteristics on the three forms of job crafting. More precisely, we found a main effect of skill variety and task significance on task crafting, a main effect of skill variety on relational crafting, and a main effect of skill variety and task identity on cognitive crafting. Openness to experience only predicted task crafting. Furthermore, our congruence hypotheses were partially supported across different types of job characteristics and job crafting. That is, we detected congruence effects between openness to experience and task significance for task crafting, between openness to experience and skill variety and task significance for relational crafting, and between openness to experience and task identity for cognitive crafting. Such differential congruence effects of different job characteristics on the three forms of job crafting provide a nuanced understanding of the role of P-J fit in job crafting. When individuals with openness to experience perform a job with skill variety, they tend to engage in more task crafting. This is because task crafting involves altering the physical aspect of work, such as the scope and content of a job. Jobs with high skill variety allow more flexibility with respect to the scope that job holders can modify, thereby providing more opportunity for task crafting. In addition to skill variety, task significance was found to be a critical component of P-J fit for relational crafting, which requires reaching out to coworkers or customers. Task significance pertains to having an impact on significant others inside and outside the organization. Therefore, when performing a job with task significance, employees are likely to craft their relationships with such significant others in a more meaningful way. Finally, task identity turned out to be a job characteristic that is, when matched with openness to experience, associated with increased cognitive crafting. This may be due to the nature of task identity. According to job characteristics theory, task identity refers to the meaning and identity that a job holder attaches to his/her job. Therefore, when employees who are open to experience perform a job with task identity, they are likely to cognitively reframe the meaning of the job. These results are consistent with Tims et al.’s [24] findings demonstrating a positive link between job crafting and perceived P-J fit. However, our study even extends Tims et al.’s by assessing objective P-J fit and revealing P-J fit in terms of what job characteristic is associated with a specific form of job crafting.

The present findings have several theoretical implications. First, many past studies have identified the antecedents of job crafting. However, most of them have examined individual (e.g., personality traits) and job characteristics as independent variables; they have not investigated the effect of the fit between these two characteristics. In this regard, the present study makes a novel and significant contribution to the existing literature. The present results, which support the tenets of the traditional P-J fit theory, also offer support to the contention that the fit between individual and job characteristics has a significant effect on job crafting. When compared to past studies, this effect was tested in a more objective manner in the present investigation. Specifically, in order to overcome the limitations of the perceptual fit measurement model, which has been used in past studies on job crafting, the actual fit measurement method was used in the present study. Second, one of the limitations of past studies on job crafting is their primary focus on task crafting [9,25,60]. In contrast, the present study is significant because it examined the effect of the fit between personality traits and job characteristics on the three dimensions of job crafting. The findings proved that the fit between personality traits and job characteristics has an effect on task, relational, and cognitive crafting. This underscores the importance of examining job crafting in a more thorough manner and from diverse perspectives. In this manner, the present findings contribute to the sophistication of existing empirical literature on job crafting. Third, the present study is significant because it not only simultaneously examined personality traits and job characteristics as workplace antecedents, but also verified the effect of the fit between these factors (i.e., interactions) on job crafting. Many scholars have noted that the characteristics of individuals, jobs, and the social environment should be simultaneously examined in order to accurately understand the behaviors of organizational members. This study has proven that this suggestion is also applicable to research on job crafting [57]. One of the highlights of the present findings is that, when self-reported levels of a personality trait (i.e., openness to experience) and job characteristics (i.e., skill variety, task significance, and task identity) were congruent at a higher level, it drove changes in organizational members’ jobs. The findings suggest that organizational members not only transform the physical boundaries of tasks (e.g., the amount and scope of work), but also reinterpret and redefine tasks, relationships, and the objectives of their work in a more meaningful manner. They change the manner in which they relate to their coworkers, superiors, and clients and expand the boundaries of their interactions. In other words, the findings suggest that, when a job that requires a high level of skill variety, task significance, or task identity is assigned to a member who is open to new experiences, imaginative, inquisitive, and an intellectual, he or she is likely to engage in job crafting more intensely, thereby creating changes in the manner in which a task is performed. This suggests that job crafting can be maximized by ensuring that the demands of individuals’ personality traits and resources that are offered by a job are congruent at a high level. Similar to past studies that have identified the antecedents of job crafting [28,60] and tested the three-factor interaction between a proactive personality, job autonomy, and interpersonal feedback [9], the present results also contribute to the existing literature on job crafting.

### 4.2. Practical Implications

In addition to these theoretical implications, the present results also have practical implications. Initiative and autonomy have been identified as key factors that help individuals cope with the demands of rapidly changing business environments in a flexible manner. Consequently, there has also been an increase in the importance that is ascribed to job crafting, which is a bottom-up approach to job redesign that involves active involvement in one’s job. This trend may be attributable to the fact that top-down approaches to job (re)design are inappropriate in contemporary work environments. The results of the present study, which comprehensively investigated the effect of the fit between openness to experience and skill variety, task significance, and task identity, suggest that appropriate conditions and resources should be provided (i.e., job characteristics) to members to encourage them to voluntarily engage in job crafting. Therefore, to promote job crafting among organizational members, managers should pay attention to business practices such as introducing and improving human resources and job schemes. For instance, to nurture the defining features of openness to experience (e.g., creativity, preference for challenges, and a sense of adventure), managers should improve the educational and training programs that are provided to members and try to integrate these values into the corporate culture of the organization in order to attract new talent. In addition, by avoiding the assignment of tasks that can be mindlessly performed using only basic skills, it is possible to create an environment in which members acquire the skills that are required to engage in challenging and creative work and better understand the significance of their jobs through interactions with superiors or coworkers. Members should be frequently afforded the opportunity to complete a task from start to finish. In other words, practical efforts should be taken to restructure jobs or the human resources system.

### 4.3. Limitations and Suggestions for Future Research

Despite the theoretical and practical implications of its findings, the present study has a few limitations. First, because the study used a cross-sectional research design, inferences about the causality of the emergent relationships cannot be drawn. The directionality of the emergent relationships cannot be ascertained because all the study variables (i.e., independent and dependent) were measured at the same time. Therefore, it may be argued that employees able to implement job crafting strategies can also enhance the level of skill variety, task significance, and task identity as an attempt to shape a job that is more consistent with their personal characteristics and preferences. To resolve this causality issue, future studies should adopt longitudinal research designs (e.g., repeated measures and lagged effects) and employ different time points to test the effect of the fit between openness to experience and job characteristics (i.e., skill variety, task significance, and task identity) on job crafting. Moreover, the proposed causality between P-J fit and job crafting can be better determined in experimental research. That is, future researchers may want to undertake an experiment in which participants’ job characteristics are manipulated to assess the effect of the degree of fit between openness to experience and job characteristics on job crafting. Although such a research design may not be feasible in real organizational settings, future experiments can be implemented for college students.

Second, we examined openness to experience, job characteristics, and job crafting within the context of a team project that was assigned to students, which limits the generalizability of our findings. To address these limitations, future studies should use samples of workers who occupy different organizational positions and represent diverse industries to test the validity of the present findings on the effect of organizational members’ openness to experience, job characteristics, and the fit between these two characteristics on job crafting.

Finally, this study investigated the effect of the fit between openness to experience and three job characteristics on job crafting. As our research examined objective P-J fit (i.e., separate measurement of the person and job characteristics) through polynomial regression, we could not include many variables in the regression equation due to the power issue, which prevented us from assessing potential mediation or moderation affecting the relationship between P-J fit and job crafting. Although subjective P-J fit (i.e., direct assessment of the extent to which the target person perceives a high level of fit between his/her needs and abilities and job characteristics) is good at exploring the mediators and moderators of P-J fit [24], it is vulnerable to rater biases and common method variance. Moreover, subjective P-J fit cannot provide knowledge of P-J fit in terms of which aspect is critical to a specific form of job crafting. For this reason, we chose a simplified P-J fit model in favor of the benefits of polynomial regression. However, the question of how the fit between openness to experience and the three job characteristics promotes job crafting and what conditions influence these relationships remain unanswered in our research. With the aim of working toward a more refined understanding of personality, job characteristics, and job crafting, we call for follow-up research that explores the intermediary mechanisms (e.g., perceived meaningfulness) and boundary conditions that influence the relationship between P-J fit and job crafting.

## 5. Conclusions

The current study aimed at examining the effect of congruence between openness to experience and job characteristics on job crafting by using survey data from college students in team settings. Our findings contribute to the job crafting literature and provide novel and meaningful insights into the differential relationship between fit between openness to experience and core job characteristics and different forms of job crafting. The present findings need to be validated in organizational teams. Furthermore, future investigations into potential mediators and moderators of the relationship between P-J fit and job crafting will enrich what has been found in the present study.

## Figures and Tables

**Table 1:** Items assessing openness to experience, job characteristics, and job crafting.

**Openness to experience** I am interested in abstract ideas. I have a vivid imagination. I have a good imagination. **Job characteristics** *Skill variety* My role requires a diverse set of complex skills that are necessary to complete the team assignment. My role requires me to possess various types of knowledge that are necessary to complete the team assignment. My role requires me to possess a wide range of abilities that are necessary to complete the team assignment. My role requires me to perform a variety of tasks that are necessary to complete the team assignment. *Task significance* My role bears significance to the successful completion of the team assignment. My role has a significant impact on other team members. My role helps me enhance my knowledge and experiences. *Task identity* The objectives and outcomes of my role are clearly delineated when we work on the team assignment. My role involves completing a task that has an obvious beginning and end when we work on the team assignment. My role allows me to complete the work that I start. **Job crafting** *Cognitive crafting* I have thought about the kind of positive impact that my role in the group assignment will have on my college life. I have reminded myself about the importance of my role in the group assignment to the larger community. I have reflected upon the effect that my role in the group assignment has on my overall well-being. *Task crafting* I have adopted a new approach to get the assignment done well. I have changed the scope or types of tasks that I complete as a part of my role in the team assignment. I have introduced new tasks that I think are better suited to my skills and interests. I have chosen to undertake additional responsibilities that pertain to the group assignment. *Relational crafting* I have tried to organize or actively participate in social activities and meetings that are related to the team assignment. I have made an effort to get to know other members of my team. I have tried to organize special events for my team members (e.g., celebrating a team member’s birthday). I have tried to mentor my friends who work with me on the group assignment. I have tried to befriend team members with similar skills and interests

**Table 2:** Means, standard deviations, and intercorrelations between the study variables.

Variable	M	SD	1	2	3	4	5	6
1. Openness to experience	3.38	0.86	-					
2. Skill variety	3.37	0.74	0.29 ***	-				
3. Task significance	3.83	0.72	0.16 *	0.41 ***	-			
4. Task identity	3.72	0.69	0.19 *	0.41 ***	0.50 ***	-		
5. Task crafting	3.38	0.68	0.37 ***	0.52 ***	0.41 ***	0.34 ***	-	
6. Relational crafting	3.26	0.80	0.16 *	0.38 ***	0.28 ***	0.28 ***	0.42 ***	-
7. Cognitive crafting	3.24	0.83	0.18 *	0.40 ***	0.17 *	0.30 ***	0.43 ***	0.41 ***

* *p* < 0.05, ** *p* < 0.01, *** *p* < 0.001.

**Table 3:** Results of polynomial regression analysis for task crafting and the response surface test.

Variable		Task Crafting				
		Model 1	Model 2	Model 3	Model 4	Model 5
*Control variables*						
Class dummy 1		−0.06	−0.03	−0.01	−0.07	−0.07
Class dummy 2		−0.04	−0.03	−0.02	−0.05	−0.09
Grade dummy 1		0.06	0.12	0.14	0.12	0.07
Grade dummy 2		−0.05	0.04	0.06	0.04	−0.03
Grade dummy 3		0.08	0.13	0.14	0.10	0.08
*Main effect*						
Openness to experience (OE)			0.21 **			
Skill variety (SV)			0.35 ***			
Task significance (TS)			0.19 **			
Task identity (TI)			0.06			
*Polynomial terms*						
OE				0.21 **		
SV				0.48 ***		
OE^2^				0.02		
OE × SV				−0.10		
SV^2^				0.07		
OE					0.26 **	
TS					0.38 ***	
TS^2^					−0.12+	
OE × TS					0.08	
TS^2^					0.00	
OE						0.29 ***
TI						0.30 ***
OE^2^						−0.06
OE × TI						0.02
TI^2^						0.00
F		0.45	11.32	8.80	6.66	4.97
R^2^		0.01	0.38	0.34	0.28	0.23
Change in R^2^			0.37 ***	0.33 ***	0.27 ***	0.21 ***
Surface response test						
Y = X	Slope (a_1_ = b_1_ + b_2_)			0.61 ***	0.56 ***	0.52 ***
	Curvature (a_2_ = b_3_ + b_4_ + b_5_)			−0.02	0.01	−0.01
Y = −X	Slope (a_3_ = b_1_ − b_2_)			−0.28 *	−0.15	−0.07
	Curvature (a_4_ = b_3_ − b_4_ + b_5_)			0.19	−0.16+	−0.07

+ *p* < 0.10, * *p* < 0.05, ** *p* < 0.01, *** *p* < 0.001.

**Table 4:** Results of polynomial regression analysis for relational crafting and the response surface test.

Variable		Relational Crafting				
		Model 1	Model 2	Model 3	Model 4	Model 5
*Control variables*						
Class dummy 1		−0.09	−0.06	−0.06	−0.09	−0.08
Class dummy 2		0.04	0.03	0.04	0.00	0.00
Grade dummy 1		−0.09	−0.05	−0.03	−0.03	−0.07
Grade dummy 2		−0.05	−0.00	0.01	−0.02	−0.05
Grade dummy 3		−0.09	−0.05	−0.04	−0.06	−0.07
*Main effect*						
Openness to experience (OE)			0.05			
Skill variety (SV)			0.27 **			
Task significance (TS)			0.11			
Task identity (TI)			0.08			
*Polynomial terms*						
OE				0.09		
SV				0.32 ***		
OE^2^				−0.09		
OE × SV				0.18 *		
SV^2^				−0.15		
OE					0.04	
TS					0.27 ***	
TS^2^					−0.15+	
OE × TS					0.20 *	
TS^2^					−0.02	
OE						0.11
TI						0.26 **
OE^2^						−0.07
OE × TI						0.04
TI^2^						−0.04
F		0.64	4.26	4.08	3.08	2.13
R^2^		0.01	0.18	0.19	0.15	0.09
Change in R^2^			0.17 ***	0.18 ***	0.14 ***	0.08 **
Surface response test						
Y = X	Slope (a_1_ = b_1_ + b_2_)			0.44 ***	0.34 **	0.41 ***
	Curvature (a_2_ = b_3_ + b_4_ + b_5_)			−0.02	0.12	−0.06
Y = −X	Slope (a_3_ = b_1_ − b_2_)			−0.26 *	−0.26 *	−0.02
	Curvature (a_4_ = b_3_ − b_4_ + b_5_)			−0.46 **	−0.44 **	−0.19

+ *p* < 0.10, * *p* < 0.05, ** *p* < 0.01, *** *p* < 0.001.

**Table 5:** Results of polynomial regression analysis for cognitive crafting and the response surface test.

Variable		Cognitive Crafting				
		Model 1	Model 2	Model 3	Model 4	Model 5
*Control variables*						
Class dummy 1		0.00	0.03	0.04	−0.01	0.02
Class dummy 2		0.04	0.02	0.06	0.02	0.02
Grade dummy 1		−0.13	−0.09	−0.09	−0.11	−0.08
Grade dummy 2		−0.01	−0.02	0.03	0.00	0.00
Grade dummy 3		−0.15	−0.10	−0.12	−0.15	−0.11
*Main effect*						
Openness to experience (OE)			0.07			
Skill variety (SV)			0.32 ***			
Task significance (TS)			−0.05			
Task identity (TI)			0.17 *			
*Polynomial terms*						
OE				0.11		
SV				0.39 ***		
OE^2^				0.13		
OE × SV				−0.11		
SV^2^				−0.07		
OE					0.15+	
TS					0.14+	
TS^2^					0.01	
OE × TS					0.08	
TS^2^					0.00	
OE						0.17 *
TI						0.27 ***
OE^2^						−0.01
OE × TI						0.11
TI^2^						−0.21 **
F		0.61	4.65	4.22	1.60	3.45
R^2^		0.01	0.20	0.20	0.08	0.17
Change in R^2^			0.19 ***	0.19 ***	0.07 *	0.16 ***
Surface response test						
Y = X	Slope (a_1_ = b_1_ + b_2_)			0.55 ***	0.55 ***	0.50 ***
	Curvature (a_2_ = b_3_ + b_4_ + b_5_)			−0.11	−0.1	−0.17
Y = −X	Slope (a_3_ = b_1_ − b_2_)			−0.33 **	−0.33 **	−0.16
	Curvature (a_4_ = b_3_ − b_4_ + b_5_)			0.16	0.16	−0.50 **

+ *p* < 0.10, * *p* < 0.05, ** *p* < 0.01, *** *p* < 0.001.

## References

[1] Grant A.M., Ashford S.J. (2008). The dynamics of proactivity at work. Res. Organ. Behav..

[2] Griffin M.A., Neal A., Parker S.K. (2007). A new model of work role performance: Positive behavior in uncertain and interdependent contexts. Acad. Manag. J..

[3] Tims M., Bakker A.B., Derks D. (2013). The impact of job crafting on job demands, job resources, and well-being. J. Occup. Health Psychol..

[4] Nielsen K., Abidgaard J.S. (2012). The development and validation of a job crafting measure for use with blue-collar worker. Work Stress.

[5] Tims M., Bakker A.B., Derks D., van Rhenen W. (2013). Job crafting at the team and individual level: Implications for work engagement and performance. Group Organ. Manag..

[6] Demerouti E., Bakker A.B., Peeters M.C.W., de Jonge J., Taris T.W. (2013). Job crafting. An Introduction to Contemporary Work Psychology.

[7] Kim S.Y., Bae S.H., Kim H.G., Ahn S.I. (2016). The effect of job crafting behavior on innovative behavior focused on mediating effect of work engagement. Korean J. Resour. Dev..

[8] Roczniewska M., Bakker A.B. (2016). Who seeks job resources, and who avoids job demands? The link between dark personality traits and job crafting. J. Psychol..

[9] Shin I.Y. (2015). Main and interaction effects of determinants in individual, job, and relational aspects on job crafting. Korean Corp. Manag. Rev..

[10] Mazzetti G., Lancioni C., Derous E., Guglielmi D. (2018). Tackling job insecurity: Can a boundaryless career orientation boost job crafting strategies and career competencies?. Psicol. Soc..

[11] Kristof A.K. (1996). Person-organization fit: An integrative review of its conceptualization, measurement, and implication. Pers. Psychol..

[12] Schneider B., Smith D.B., Goldstein H.W. (2000). Attraction-Selection-Attrition: Toward a Person-Environment Psychology of Organizations.

[13] Edwards J.R. (1991). Person-Job Fit: A Conceptual Integration, Literature Review, and Methodological Critique.

[14] Baer M., Oldham G.R. (2006). The curvilinear relation between experienced creative time pressure and creativity: Moderating effects of openness to experience and support for creativity. J. Appl. Psychol..

[15] Ivcevic Z., Brackett M. (2014). Predicting school success: Comparing conscientiousness, grit, and emotion regulation ability. J. Res Personal..

[16] Madrid H.P., Patterson M.G. (2016). Creativity at work as a joint function between openness to experience, need for cognition and organizational fairness. Learn Individ. Differ..

[17] Simmons A.L. (2011). The influence of openness to experience and organizational justice on creativity. Creat. Res J..

[18] Tan C.S., Lau X.S., Kung Y.T., Kailsan R.A.L. (2019). Openness to experience enhances creativity: The mediating role of intrinsic motivation and the creative process engagement. J. Creat. Behav..

[19] Xu S., Jiang X., Walsh I.J. (2018). The influence of openness to experience on perceived employee creativity: The moderating roles of individual trust. J. Creat. Behav..

[20] Zhang W., Sun S.L., Jiang Y., Zhang W. (2019). Openness to Experience and Team Creativity: Effects of Knowledge Sharing and Transformational Leadership. Creat. Res. J..

[21] McCrae R.R., Costa Jr P.T., Del Pilar G.H., Rolland J.P., Parker W.D. (1998). Cross-cultural assessment of the five-factor model: The Revised NEO Personality Inventory. J. Cross Cult. Psychol..

[22] Hackman J.R., Oldham G.R. (1976). Motivation through the design of work: Test of a theory. Organ. Behav. Hum. Perform..

[23] Lee S.H., Shin Y., Kim M. (2018). Why work meaningfulness alone is not enough: The role of social identification and task interdependence as facilitative boundary conditions. Curr. Psychol..

[24] Tims M., Derks D., & Bakker A.B. (2016). Job crafting and its relationships with person-job fit and meaningfulness: A three-wave study. J. Vocat. Behav..

[25] Wrzesniewski A., Dutton J.E. (2001). Crafting a job: Revisioning employees as active crafters of their work. Acad. Manag. Rev..

[26] Tims M., Bakker A.B. (2010). Job crafting: Towards a new model of individual job redesign. SA J. Ind. Psychol..

[27] Slemp G.R., Vella-Brodrick D.A. (2013). The job crafting questionnaire: A new scale to measure the extent to which employees engage in job crafting. Int. J. Wellbeing.

[28] Lim M.K., Ha Y.J., Oh D.J., Sohn Y.W. (2014). Validation of the Korean version of Job Crafting Questionnaire (JCQ-K). Korean Corp. Manag. Rev..

[29] Wrzesniewski A., LoBuglio N., Dutton J.E., Berg J.M. (2013). Job crafting and cultivating positive meaning and identity in work. Adv. Posit. Organ. Psychol..

[30] Hildenbrand K., Scaramento C.A., Binnewies C. (2018). Transformational leadership and burnout: The role of thriving and followers’ openness to experience. J. Occup. Health Psychol..

[31] Costa P.R., McCrae R.R. (1985). The NEO Personality Inventory.

[32] George J.M., Zhou J. (2001). When openness to experience and conscientiousness are related to creative behavior: An interactional approach. J. Appl. Psychol..

[33] Halbesleben J.R., Wheeler A.R. (2011). I owe you one: Coworker reciprocity as a moderator of the day-level exhaustion-performance relationship. J. Organ. Behav..

[34] Ten Brummelhuis L.L., Bakker A.B. (2012). A resource perspective on the work-home interface: The work-home resource model. Am. Psychol..

[35] LePine J.A., Colquitt J.A., Erez A. (2000). Adaptability to changing task contexts: Effects of general cognitive ability, conscientiousness, and openness to experience. Pers. Psychol..

[36] Driskell J.E., Goodwin G.F., Salas E., O’Shea P.G. (2006). What makes a good team player? Personality and team effectiveness. Group Dyn. Theory Res. and Pract..

[37] Williams S., Shiaw W.T. (1999). Mood and organizational citizenship behavior: The effects of positive affect on employee organizational citizenship behavior intention. J. Psychol..

[38] Hackman J.R., Oldham G.R. (1980). Work Redesign.

[39] Bakker A.B., Demerouti E., Euwema M.C. (2005). Job resources buffer the impact of job demands on burnout. J. Occup. Health Psychol..

[40] Conger J.A., Kanungo R.N. (1988). The empowerment process: Integrating theory and practice. Acad. Manag. Rev..

[41] Shim D., Yang D., Ha S. (2010). The mediating effects of psychological empowerment in the relationship between job characteristics, locus of control and leader-member exchange, and job performance. Korean J. Manag..

[42] French J.R., Caplan R.D., Van Harrison R. (1982). The Mechanisms of Job Stress and Strain.

[43] Sung J.Y., Park W.-W., Yoon S. (2008). The effect of person-environment (organization, supervisor and coworker) fit, on organizational citizenship behavior and performance, and the mediating effect of justice. Korean J. Manag..

[44] Maslach C., Leiter M.P. (1997). The Truth about Burnout: How Organizations Cause Personal Stress and What to do About It.

[45] Leiter M.P., Maslach C. (1999). Six areas of worklife: A model of the organizational context of burnout. J. Health Hum. Serv. Adm..

[46] Brislin R.W., Lonner W.J., Berry J.W. (1986). The wording and translation of research instruments. Field Methods in Cross-Cultural Research.

[47] Costello A.B., Osborne J.W. (2005). Best practices in exploratory factor analysis: four recommendations for getting the most from your analysis. Pract. Assess. Res. Eval..

[48] Brown T.A. (2006). Confirmatory factor analysis for applied research.

[49] Alderdice F., Savage-McGlynn E., Martin C., McAuliffe F., Hunter A., Unterscheider J., Daly S., Geary M., Kennelly M., O’Donoghue K. (2013). The Prenatal Distress Questionnaire: An investigation of factor structure in a high risk population. J. Reprod. Infant Psychol..

[50] Picco L., Abdin E., Chong S.A., Pang S., Shafie S., Chua B.Y., Subramaniam M. (2016). Attitudes toward seeking professional psychological help: Factor structure and socio-demographic predictors. Front. Psychol..

[51] Donnellan M.B., Oswald F.L., Baird B.M., Lucas R.E. (2006). The mini-IPIP scales: Tiny-yet effective measures of the Big Five factors of personality. Psychol. Assess..

[52] Donnellan M.B., Lucas R.E. (2008). Age differences in the big five across the life span: evidence from two national samples. Psychol. Aging.

[53] Grant A.M., Berry J.W. (2011). The necessity of others is the mother of invention: Intrinsic and prosocial motivations, perspective taking, and creativity. Acad. Manag. J..

[54] Credé M., Harms P., Niehorster S., Gaye-Valentine A. (2012). An evaluation of the consequences of using short measures of the Big Five personality traits. J. Pers. Soc. Psychol..

[55] Hittner J.B., Penmetsa N., Bianculli V., Swickert R. (2020). Personality and substance use correlates of e-cigarette use in college students. Person. Individ. Differ..

[56] Morgeson F.P., Humphrey S.E. (2008). The Work Design Questionnaire (WDQ): Developing and validating a comprehensive measure for assessing job design and the nature of work. J. Appl. Psychol..

[57] Federici E., Boon C., Den Hartog D.N. (2019). The moderating role of HR practices on the career adaptability-job crafting relationship: A study among employee-manager dyads. Int. J. of Hum. Resour. Manag..

[58] Edwards J.R., Parry M.E. (1993). On the use of polynomial regression equations as an alternative to difference scores in organizational research. Acad. Manag. J..

[59] Edwards J.R., Cable D.M. (2009). The value of value congruence. J. Appl. Psychol..

[60] Leana C., Appelbaum E., Shevchuk I. (2009). Work process and quality of care in early childhood education: The role of job crafting. Acad. Manag. J..

